# Deep learning prediction of MSI/dMMR, EBV, HER2, PD-L1, and CLDN18.2 status directly from hematoxylin and eosin-stained slides in gastric and gastroesophageal junction adenocarcinoma: a systematic review

**DOI:** 10.3389/fonc.2026.1953562

**Published:** 2026-09-09

**Authors:** Hyuk Lee, Sang Yun Ha, Sehun Kim, Yang Won Min

**Affiliations:** 1 Department of Medicine, Samsung Medical Center, Sungkyunkwan University School of Medicine, Seoul, Republic of Korea; 2 Department of Pathology and Translational Genomics, Samsung Medical Center, Sungkyunkwan University School of Medicine, Seoul, Republic of Korea; 3 Samsung Precision Genome Medicine Institute, Samsung Medical Center, Seoul, Republic of Korea

**Keywords:** clinical validation, computational pathology, deep learning, diagnostic accuracy, gastric adenocarcinoma, gastroesophageal junction adenocarcinoma, hematoxylin and eosin, molecular biomarkers

## Abstract

**Background:**

Microsatellite instability (MSI)/mismatch-repair deficiency (dMMR), Epstein–Barr virus (EBV), human epidermal growth factor receptor 2 (HER2), programmed death-ligand 1 (PD-L1), and claudin 18 isoform 2 (CLDN18.2) are clinically relevant biomarkers in gastric and gastroesophageal junction (GEJ) adenocarcinoma, but molecular and immunohistochemical (IHC) testing is costly and not universally available. Deep learning (DL) applied to routine hematoxylin and eosin (H&E) slides may enable inexpensive pre-screening. We assessed the accuracy of DL models predicting these five biomarkers directly from H&E.

**Methods:**

We searched PubMed, Embase, Cochrane CENTRAL, and Web of Science Core Collection from inception through 8 August 2026 for studies applying DL to H&E whole-slide or tissue-microarray images to predict MSI/dMMR, EBV, HER2, PD-L1, or CLDN18.2 status in gastric/GEJ adenocarcinoma. Two reviewers independently selected studies and re-extracted performance estimates from full texts, tables, footnotes, and supplementary materials. Risk of bias was assessed with Quality Assessment of Diagnostic Accuracy Studies 2 (QUADAS-2) and Prediction model Risk Of Bias ASsessment Tool (PROBAST) and certainty was assessed with Grading of Recommendations Assessment, Development and Evaluation (GRADE). Because the requirements for independent, variance-supported area under the receiver-operating-characteristic curve (AUROC) estimates or threshold-specific 2 × 2 data were not met, study-level estimates were displayed without statistical pooling.

**Results:**

Of 1,094 records identified, 701 unique records were screened and 28 studies were included: 18 addressed MSI/dMMR, 14 addressed EBV, 2 addressed HER2, 2 addressed PD-L1, and 1 addressed CLDN18.2; marker categories overlapped. External-independent AUROCs were 0.767 (95% CI, 0.726–0.830) for MSI/dMMR, 0.941 (0.920–0.970) and 0.859 (0.823–0.919) for EBV, and 0.856 (0.775–0.926) for CLDN18.2; broader held-out AUROCs were 0.760 and 0.880 (MSI/dMMR) and 0.980 (EBV). One report each provided eligible 2 × 2 data for EBV, MSI/dMMR, and CLDN18.2; none did for HER2 or PD-L1. No pooling or final-model calibration was supported. All 28 reports (42 analytic scopes) were high risk under PROBAST; QUADAS-2 risk was high for 40 scopes and unclear for 2. Certainty was very low for MSI/dMMR, EBV, HER2, and PD-L1 and low for CLDN18.2.

**Conclusion:**

Individual held-out models sometimes showed promising discrimination, particularly for EBV and MSI/dMMR, but the evidence was too sparse, dependent, and heterogeneous to define a stable summary performance or a clinically deployable triage threshold. Certainty was very low for MSI/dMMR, EBV, HER2, and PD-L1 and low for CLDN18.2; clinical utility and calibrated threshold performance remain unestablished.

**Systematic review registration:**

https://www.crd.york.ac.uk/PROSPERO/, identifier CRD420261449992.

## 1 Introduction

Gastric and gastroesophageal junction (GEJ) adenocarcinoma remains a leading cause of cancer-related death worldwide, and management is increasingly biomarker-driven. Microsatellite instability-high/mismatch-repair deficiency (MSI/dMMR), human epidermal growth factor receptor 2 (HER2), programmed death-ligand 1 (PD-L1), and claudin 18 isoform 2 (CLDN18.2) can inform systemic-treatment selection, including immune checkpoint inhibition, anti-HER2 therapy, and CLDN18.2-targeted therapy (1, 2). EBV is a biologically and prognostically relevant molecular subtype and small clinical series have suggested immunotherapy-response enrichment (3), but EBV is not currently an established stand-alone treatment-selection biomarker comparable with MSI/dMMR, HER2, PD-L1, or CLDN18.2. Determining these biomarkers requires molecular assays together with immunohistochemistry (IHC) or *in situ* hybridization, which are costly, tissue-consuming, and not universally available.

Computational pathology using deep learning (DL) can infer molecular features directly from routine hematoxylin and eosin (H&E)-stained images. Since the demonstration that MSI can be predicted from histology in gastrointestinal cancer (4), numerous models have targeted individual biomarkers in gastric cancer (5–7). Because H&E slides are generated for virtually every resected or biopsied tumor at negligible marginal cost, an accurate H&E-based classifier could serve as an inexpensive, universally available pre-screen that triages which tumors proceed to confirmatory molecular or IHC testing.

Prior evidence syntheses have largely focused on colorectal MSI or imaging radiomics rather than H&E-based biomarker prediction in gastric cancer (8–10). We therefore systematically reviewed DL models predicting MSI/dMMR, EBV, HER2, PD-L1, or CLDN18.2 from gastric/GEJ H&E slides. We distinguished the volume of reported performance results from independent evaluation units and examined how validation setting, analysis unit, reference-standard validity, threshold timing, and cohort or model dependence altered the evidence display.

## 2 Methods

### 2.1 Protocol and registration

The review was registered in PROSPERO (CRD420261449992) and is reported in accordance with the Preferred Reporting Items for Systematic Reviews and Meta-Analyses (PRISMA) 2020 (11) and PRISMA-DTA (12) statements. The protocol prespecified MSI/dMMR, EBV, HER2, and PD-L1; following peer review, CLDN18.2 was added through an amendment incorporated into the PROSPERO record. Each biomarker was synthesized separately to avoid pooling across heterogeneous molecular targets.

### 2.2 Eligibility criteria

We included studies that enrolled patients with gastric or GEJ adenocarcinoma; used artificial intelligence (AI)/DL applied to H&E-stained whole-slide or tissue-microarray images as the index test; predicted MSI/dMMR, EBV, HER2, PD-L1, or CLDN18.2 status; and used a molecular, IHC, or in situ hybridization reference standard. Formal evidence synthesis was restricted to peer-reviewed full-text reports because cohort composition and independence, reference standards, validation design, threshold timing, and extractable performance data had to be verifiable. Conference abstracts and preprints were used only for subsequent-publication or companion-report tracing and never contributed directly to formal synthesis; when a linked eligible peer-reviewed full-text primary report existed, only that full-text report contributed. We excluded non-H&E inputs, AI-assisted IHC scoring, treatment-response or prognosis outcomes rather than biomarker status, molecular/omics-only or blood-based approaches, non-gastric results, and multimodal index tests requiring molecular input for the primary prediction.

### 2.3 Information sources and search

PubMed, Embase, Cochrane CENTRAL, and Web of Science Core Collection were searched from inception, with updates completed in August 2026, using concept blocks combining population (gastric/GEJ), index test (deep or machine learning and histopathology/whole-slide/H&E), and target biomarkers (MSI/dMMR, EBV, HER2/ERBB2, PD-L1/CD274, and CLDN18.2). No language or publication-date restrictions were applied. The complete executed queries, platforms, dates, hit counts, and handling decisions are provided in **Supplementary Material 1**.

### 2.4 Study selection and data extraction

Two reviewers (Y.W.M. and H.L.) independently screened 701 unique records and assessed full texts, with disagreements resolved by a third reviewer (S.Y.H.). For each study, we extracted the design, cohorts, participant/specimen and analysis units, validation setting, model architecture, encoder and pretraining source, reference standard, area under the receiver-operating-characteristic curve (AUROC) and sampling uncertainty, and every exact or uniquely recoverable threshold-specific 2 × 2 table. Extraction was based on the full text, tables, footnotes, figures, and **Supplementary Material**. All eligible estimates were retained. For synthesis, AUROC and threshold-specific DTA outcomes were routed separately using an outcome-blind hierarchy: marker-specific, H&E-only, patient- or specimen-level estimates from independent or external validation were preferred; shared models, repeated or overlapping cohorts, The Cancer Genome Atlas (TCGA) reuse, alternative thresholds, and non-primary configurations were assigned to dependence-aware, sensitivity-only, narrative, or out-of-scope strata rather than counted as independent. The complete study-level extraction, risk-of-bias, and certainty dataset is provided in **Supplementary Material 2**.

### 2.5 AI-assisted workflow support

Generative AI tools (Claude, Anthropic; Codex, OpenAI) supported spreadsheet reconciliation, consistency checks, and drawing figures. They did not replace human review: study eligibility, extracted values, risk-of-bias and Grading of Recommendations Assessment, Development and Evaluation (GRADE) judgments, and synthesis decisions were verified against the source reports and approved by the authors. The authors take full responsibility for the final content.

### 2.6 Risk-of-bias assessment

Two reviewers independently assessed each study-marker evaluation using Quality Assessment of Diagnostic Accuracy Studies 2 (QUADAS-2) (13) and Prediction model Risk Of Bias ASsessment Tool (PROBAST) (14). Development/resampling, internal held-out or shift, and external-validation layers were assessed separately. Three CLDN18.2 scopes from one report (15) and two EBV scopes from another report (16) produced 42 analytic scopes across 28 reports. Each displayed estimate was linked to the corresponding analytic-scope record and domain-level rationale in **Supplementary Material 2**. A report-level worst-domain summary was retained only as a secondary overview; overall risk was high if any domain was high.

### 2.7 Certainty of evidence

Certainty for each marker was rated with GRADE for test accuracy (17–19). We considered risk of bias, indirectness, inconsistency, imprecision, and publication bias. Because no valid pooled summary and too few independent patient-level 2 × 2 units existed, inconsistency and publication bias could not be formally rated; non-assessability was not treated as absence of concern. Clinical consequences per 1,000 patients were not calculated.

### 2.8 Protocol deviations

Relative to the registered protocol, Scopus and IEEE Xplore were not searched because institutional access was unavailable; Web of Science Core Collection and targeted PubMed recall searches were used instead. Stand-alone preprint-server searches could not be documented, and formal synthesis was restricted to peer-reviewed full texts. CLDN18.2 was added after peer review through a protocol amendment. Planned subgroup and meta-regression analyses and author queries were not undertaken because independent evidence units were sparse. The estimate-routing hierarchy was finalized during full-text re-extraction rather than prospectively and was based on design and dependence rather than performance magnitude.

### 2.9 Data synthesis

The unit of synthesis was a report-marker-cohort-model evaluation, with dependence tracked explicitly. The outcome-blind routing algorithm proceeded through eligibility, analysis unit and patient separation, validation setting, reference-standard class, metric verifiability, threshold timing, cohort/model overlap, and fixed tie-breakers favoring greater externality, patient-level evaluation, a clinically anchored reference standard, no target-test influence, the report’s primary configuration, and then larger sample size. Observed performance magnitude was not used for routing. Within each report-marker, one representative estimate was selected. Scenario A admitted genuinely external, patient-level, clinically anchored evaluations without target-cohort adaptation. Scenario B additionally admitted same-institution patient-disjoint held-out evaluations. Test-influenced, surrogate/unclear-reference, cohort-specific Youden, and overlapping estimates were retained only in labeled secondary, sensitivity, dependence-aware, or narrative strata. We did not pool AUROCs or sensitivity/specificity because no marker provided enough independent estimates from a common clinical stratum. Author-reported AUROCs and confidence intervals were displayed without pooling; exact Clopper-Pearson intervals were calculated from 2 × 2 counts.

## 3 Results

### 3.1 Study selection

Database searches and updates identified 1,094 records. After 393 duplicates were removed, 701 unique records were screened and 664 were excluded at title/abstract. All 37 reports sought for retrieval were obtained and assessed; 9 were excluded after full-text review (wrong outcome/target condition, 5; wrong index test, 2; wrong population/cancer site, 1; no extractable gastric/GEJ performance, 1). Twenty-eight studies were included (**Figure 1**).

**Figure 1:**
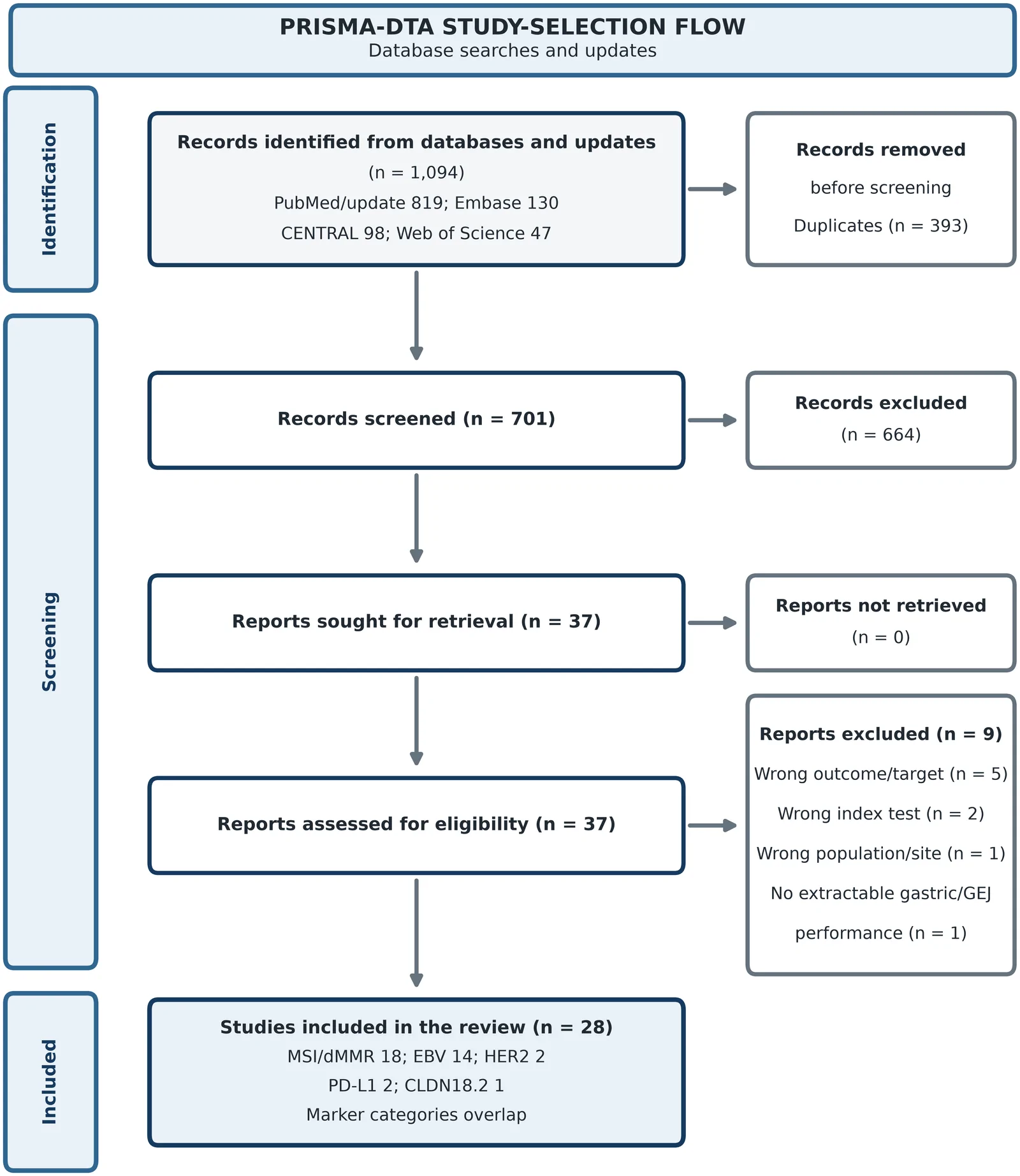
PRISMA-DTA flow diagram. Database searches and updates identified 1,094 records; 393 duplicates were removed, 701 unique records were screened, 37 full texts were assessed, 9 were excluded, and 28 studies were included. GEJ, gastroesophageal junction; MSI/dMMR, microsatellite instability or mismatch-repair deficiency.

### 3.2 Study characteristics

The 28 included studies (**Table 1**) addressed MSI/dMMR (*n* = 18) (5–7, 20–34), EBV (*n* = 14) (5–7, 16, 20, 21, 31, 33–39), PD-L1 (*n* = 2) (7, 40), HER2 (*n* = 2) (41, 42), and CLDN18.2 (*n* = 1) (15); markers overlapped. The supplementary workbook contains 204 AUROC rows and 40 exact or uniquely recoverable threshold-specific 2 × 2 rows; some describe the same model-cohort configuration, and neither total represents independent evidence. Cohorts were mainly retrospective institutional or public datasets. The CLDN18.2 study (15) included development, internal-validation, and ethnically diverse external-validation cohorts; centralized blinded IHC defined positivity as moderate-to-strong membranous staining in at least 75% of tumor cells. The EBV biopsy study (16) used gastric-biopsy H&E slides with EBER-ISH labels, 744 internal cases assessed by patient-level fivefold cross-validation, and an external 157-case institutional cohort.

**Table 1:** Characteristics of the 28 included studies.

Study (identifier)	Marker(s)	Country	Design/validation	Reference standard	Quantitative synthesis role
Jeong 2022 (PMID 36323712)	EBV	Republic of Korea; USA	Geographically/institutionally external test after domain fine-tuning	TCGA molecular EBV result/RNA-seq; institutional cohorts EBER-ISH	Threshold-sensitivity-analysis 2 × 2 result (EBV; same-test threshold)
Zheng 2022 (PMID 35589792)	EBV	China; USA	Fivefold internal evaluation split at slide level with patient separation unreported; geographically/institutionally external multicenter test; geographically external public TCGA test	Internal-STAD and multicenter-STAD: institutional EBER *in situ* hybridization; TCGA-STAD: published genetic-sequencing EBV label	External-independent AUROC (EBV); external locked-threshold 2 × 2 (EBV)
Vuong 2022 (PMID 36205993)	EBV	Republic of Korea	Independent held-out same-institution biopsy test with specimen-type shift; not external validation; 10% operating threshold selected on the same test cohort	EBER-ISH using BOND ready-to-use EBER probe/BOND III; diffuse strong nuclear positivity defined EBV-positive	Conditional same-institution specimen-shift AUROC; threshold-sensitivity-analysis 2 × 2 result (EBV; same-test threshold)
Saldanha 2023 (PMID 36264524)	MSI/dMMR, EBV	Switzerland; United Kingdom; Germany; USA	Geographically external public test cohort for all local, centralized, and swarm models	BERN/LEEDS: MSI by IHC and EBV by EBER-ISH; TUM: MSI by PCR and EBV by EBER-ISH; TCGA: molecular/genetic status	Narrative/dependence-aware
Wang 2022 (PMID 35801909)	MSI/dMMR, EBV	USA/public TCGA	Internal patient-level held-out test; not external	TCGA integrated molecular subtype labels from Bass et al.	Narrative/dependence-aware
Muti 2021 (PMID 34417147)	MSI/dMMR, EBV	Switzerland; South Korea; UK; Japan; Germany; Italy; USA	Internal patient-level CV plus geographically/institutionally external patient tests; no patient appears in training and test in the same experiment	Cohort-specific PCR or MMR IHC for MSI/dMMR (TCGA genetic test); EBER-ISH for EBV (TCGA genetic test)	External-independent AUROC (EBV, MSI/dMMR); shared-model dependence flagged
Hinata 2021 (PMID 34811485)	MSI/dMMR, EBV	Japan; USA	Internal patient-level held-out test; pure external TCGA test; separate target-fine-tuned TCGA holdout	EBER-ISH positive for EBV; loss of any MLH1/MSH2/MSH6/PMS2 by IHC for MSI/dMMR; TCGA molecular class from original TCGA report	Broader patient-disjoint held-out AUROC (EBV, MSI/dMMR)
Wei 2023 (PMID 37356573)	MSI/dMMR, EBV, PD-L1	USA/public TCGA; China	Internal random split plus geographically/institutionally external patient cohort	TCGA EBV/MSI labels from Liu et al.; TCGA PD-L1 expression from cBioPortal; FHCMU EBER-ISH for EBV and PD-L1 IHC (>1% stained cells)	Narrative/dependence-aware
Zhang 2021 (PMID 34885112)	EBV	USA/public TCGA	Internal held-out patient test from the same TCGA cohort	RNA-seq EBV abundance: EBV+ if >100 RPHM; EBV− if EBV = 0 and HHV5 = 0	Narrative/dependence-aware
Wu 2025 (PMID 39762854)	HER2	China	Internal cross-validation plus same-institution held-out biopsy test (*N* = 101)	IHC 0/1+ negative, 3+ positive; IHC 2+ resolved by FISH (HER2/CEP17 ratio ≥2 or ratio <2 with average copy number ≥6)	Narrative/dependence-aware
Liao 2025 (PMID 39792693)	HER2	China	Internal held-out WSI test plus institutionally external multicenter WSI test; external test reused for component/model selection	HER2 high expression: IHC 3+ or IHC 2+ with FISH/ISH positivity; IHC 0/1+ negative; gastric 10% immunoreactive-cell criterion	Test-set-influenced secondary 2 × 2 (HER2)
Chang 2026 (PMID 42339119)	MSI/dMMR	USA/public TCGA; China	Internal fivefold CV; external text-expanded cohort and likely preliminary figure cohort, which are overlapping rather than independent	TCGA MSI signature source not fully specified; external MSI ground truth by PCR (with NGS also performed)	Narrative/dependence-aware
Park 2025 (PMID 40389599)	MSI/dMMR	USA; South Korea; international public data	Internal WSI-level cross-validation plus inter-cohort validation; external tests are not uniformly institutionally independent and participant overlap is unquantified	TCGA-STAD: sequencing-derived MSI label; Yonsei-Classic: five-mononucleotide PCR panel; STMary-GC and GC-ICI: five-marker Bethesda PCR panel with MSI-L grouped as negative; molecular subtypes: PCR plus IHC, with the joint/discrepancy rule unreported	Narrative/dependence-aware
Lee 2025 (PMID 40953016)	MSI/dMMR	South Korea/public international	Internal patient-level held-out test	PCR MSI results from GDC	Broader patient-disjoint held-out AUROC (MSI/dMMR)
Zhang 2025 (PMID 41214872)	MSI/dMMR	China/public international	Internal cross-validation for gastric subset	TCGA MSI label; exact assay not reported in accessible text	Narrative/dependence-aware
Li 2025 (PMID 40852482)	MSI/dMMR	China	Internal random held-out slide test; patient separation not reported	Multiplex fluorescent PCR capillary electrophoresis or NGS	Narrative/dependence-aware
Murchan 2024 (PMID 39398947)	MSI/dMMR	Ireland/public international	Internal patient-level cross-validation	Clinical/genetic attributes from cBioPortal; exact MSI assay NR	Narrative/dependence-aware
Su 2022 (PMID 35177797)	MSI/dMMR	China	Primary held-out test (*N* = 88); secondary integration cohort (*N* = 31)	MMR IHC for all; cases classified as dMMR by IHC were additionally verified by PCR	Broader patient-disjoint held-out 2 × 2 (MSI/dMMR); threshold sensitivity result
Lee 2023 (PMID 36054320)	MSI/dMMR	South Korea/public international	External geographically/institutionally distinct validation for TCGA-trained FFPE model	TCGA MSI status; institutional reference assay NR	Reference-standard sensitivity analysis (MSI/dMMR)
Valieris 2020 (PMID 33316873)	MSI/dMMR	Brazil/public international	Internal patient-grouped held-out validation	Mutational Signature 20 activity as surrogate ground truth for mismatch-repair deficiency	Narrative/dependence-aware
Yasuda 2020 (PMID 32012497)	PD-L1	Japan	Internal repeated image-level cross-validation	Quantified PD-L1 IHC signal and tumor proportion score	Narrative/dependence-aware
Byeon 2025 (PMID 41062551)	EBV	South Korea/public international	Multiple same-institution scanner/specimen tests plus public external TCGA; GAT/GGT are paired duplicate scans	EBER *in situ* hybridization	Narrative/dependence-aware
Flinner 2022 (PMID 35119111)	MSI/dMMR, EBV	Germany	Geographically/institutionally external validation	TCGA molecular classes; UKC EBER-ISH for EBV and MLH1/MSH2 IHC loss for MSI	Narrative/dependence-aware
Yu 2023 (PMID NR)	MSI/dMMR	Taiwan/public international	Institutionally held-out CGMH test (87 WSIs; 49 tumor WSIs) plus TCGA within-dataset patch split; no 2 × 2 reconstructed because Figure 11 was internally inconsistent	NR	Narrative/dependence-aware
Zhao 2024 (PMID 39738468)	MSI/dMMR, EBV	China/public international	Internal cross-validation; patient separation not independently reproducible from released files	TCGA molecular subtype labels; exact assay NR	Narrative/dependence-aware
Laleh 2022 (PMID 35588568)	MSI/dMMR, EBV	Germany; Switzerland; public international	Internal patient-level CV plus external public cohort	BERN: dMMR/MSI by IHC for DNA mismatch-repair proteins and EBV by EBER *in situ* hybridization; TCGA-STAD: public MSI/EBV labels from Liu et al. (2018)	Narrative/dependence-aware
Kim 2026 (PMID 42407198)	CLDN18.2	Republic of Korea; Italy	Development/tuning, patient-disjoint internal validation, and institutionally/geographically external validation; tuning-fixed 90% sensitivity threshold transported without external adaptation	Centralized blinded CLDN18.2 IHC; positive if at least 75% of tumor cells had moderate-to-strong membranous staining	External-independent AUROC and locked-threshold 2 × 2 (CLDN18.2); internal and cohort-specific thresholds secondary
Kang 2026 (PMID 42557819)	EBV	Republic of Korea	Retrospective four-institution gastric-biopsy study; patient-level fivefold internal cross-validation and institutionally external EWUM validation; external cohort reused for model comparison/selection and threshold exploration	EBER-ISH status from pathology reports; assay, positivity criterion, blinding, and index-reference interval not reported	Internal CV narrative/dependence-aware; external AUROC and exact 2 × 2 secondary/test-influenced; same-cohort threshold 0.29 sensitivity analysis only

Repeated cohorts or shared models were not treated as independent evidence. AUROC, area under the receiver operating characteristic curve; EBV, Epstein–Barr virus; GEJ, gastroesophageal junction; MSI/dMMR, microsatellite instability or mismatch-repair deficiency; NR, not reported; PD-L1, programmed death-ligand 1.

### 3.3 Risk of bias and applicability

Across 42 analytic scopes, QUADAS-2 overall judgments were high for 40 and unclear for 2, whereas all 42 PROBAST overall judgments were high; all 28 reports were high under the secondary report-level worst-domain rule for both tools (**Table 2**). Scope-level presentation showed that development/resampling, internal held-out or shift, and external evaluation differed materially despite predominantly high overall judgments. Common concerns included sampling, small event counts, threshold or model selection using evaluation data, unclear patient separation, public or surrogate labels, incomplete flow, absent calibration, clustering, multiplicity, and possible pretraining-evaluation overlap.

**Table 2:** Analytic-scope risk-of-bias summary by evaluation setting (42 scopes).

Evaluation setting	Scopes (*n*)	QUADAS-2 overall high	PROBAST overall high	Interpretive note
Development/cross-validation/resampling	9	9	9	Model-development evidence; not an external test
Internal held-out/same-institution shift	14	13	14	Patient separation and specimen/scanner shift shown separately
External validation	19	18	19	Public versus institutionally independent and test adaptation shown separately

Each analytic scope was assessed separately; report-level worst-domain ratings were used only as a secondary overview.

### 3.4 Certainty of evidence

Certainty was very low for MSI/dMMR, EBV, HER2, and PD-L1 and low for CLDN18.2. These four markers were downgraded for very serious risk of bias together with indirectness and/or imprecision. The EBV biopsy study (16) added direct biopsy evidence but was secondary/test-influenced, had only 25 external EBV-positive cases, and did not resolve EBV risk of bias, indirectness, or imprecision. CLDN18.2 was downgraded one level for serious risk of bias and one for serious imprecision, with no downgrade for indirectness; inconsistency and publication bias were not formally ratable. Restriction to peer-reviewed full texts may have omitted emerging, null, or unfavorable findings, and non-assessability of publication bias was not evidence of its absence.

### 3.5 MSI/dMMR

MSI/dMMR evidence was reported in 18 studies. Under Scenario A, one clinically anchored external-independent report-marker line had AUROC 0.767 (95% CI, 0.726–0.830). Scenario B added two patient-disjoint internal held-out lines, including AUROC 0.880 (0.759–1.000); an external estimate of 0.874 (0.833–0.914) was retained only in the reference-standard sensitivity analysis because the institutional assay was not reported. One patient-disjoint held-out 2 × 2 evaluation used a training-selected 30.7% threshold and yielded sensitivity 0.833 (0.672–0.936) and specificity 0.885 (0.766–0.956). No summary estimate was calculated. Representative AUROC estimates under the validation-routing framework are shown in **Figure 2**.

**Figure 2:**
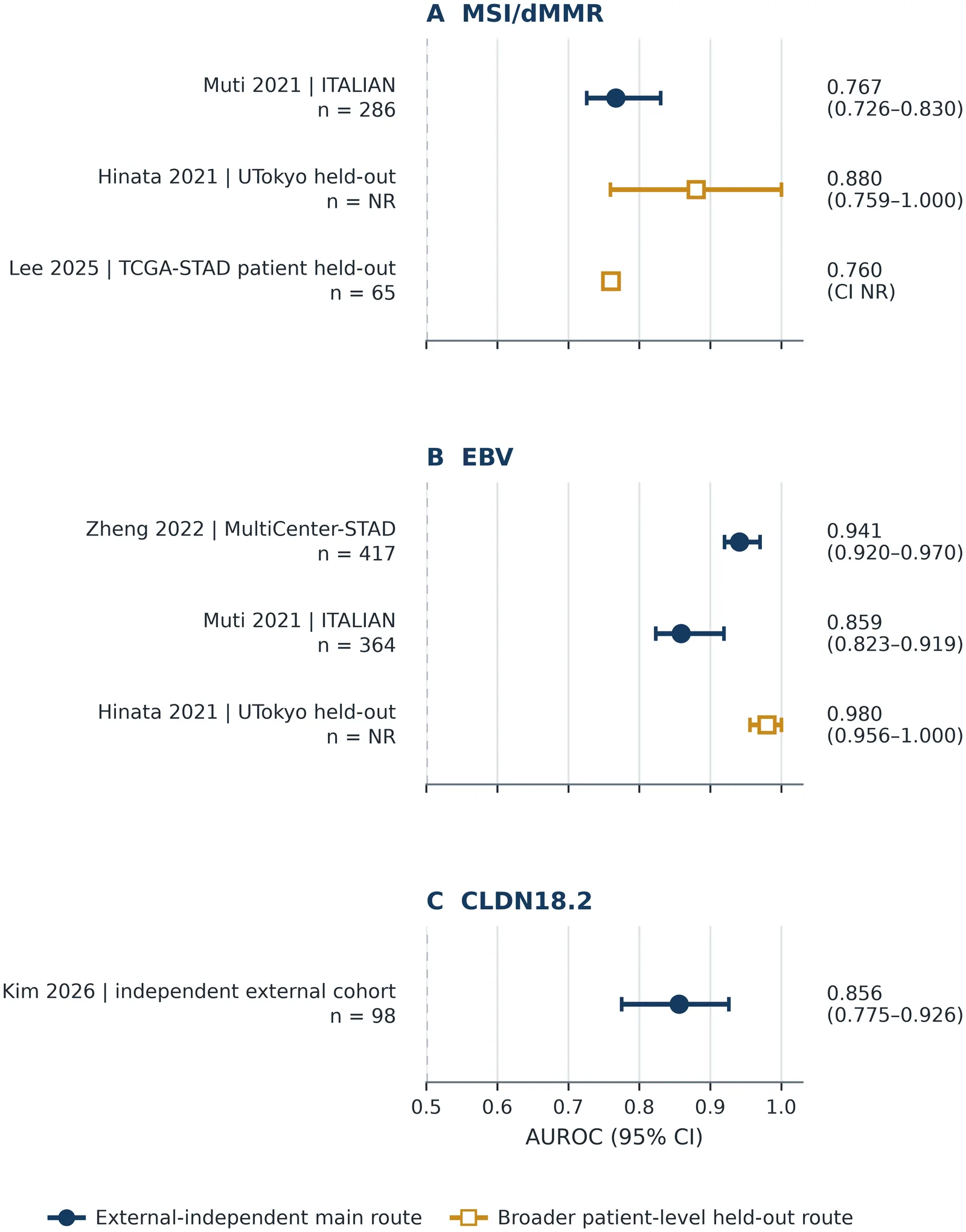
Representative AUROC estimates under the validation-routing framework: **(A)** MSI/dMMR, **(B)** EBV, and **(C)** CLDN18.2. The paired MSI/dMMR and EBV estimates from the same report (6) are not independent. No HER2 or PD-L1 AUROC met either display route; no pooling was performed. AUROC, area under the receiver operating characteristic curve; CI, confidence interval; NR, not reported.

### 3.6 EBV

EBV evidence was reported in 14 studies. EBV was analyzed as a molecular subtype, not described as an approved stand-alone companion diagnostic. Scenario A displayed two clinically anchored external-independent AUROCs: 0.941 (95% CI, 0.920–0.970) and 0.859 (0.823–0.919). Scenario B added one patient-disjoint internal held-out AUROC of 0.980 (0.956–1.000). A same-institution specimen-shift estimate of 0.872 (0.756–0.950) remained conditional because patient equivalence and threshold optimization required separate consideration. One external multicenter evaluation used a development-set threshold and yielded sensitivity 0.969 (0.913–0.994) and specificity 0.759 (0.708–0.805). In the EBV biopsy study (16), DSMIL-MB achieved internal cross-validation AUROC 0.907 ± 0.044 and external AUROC 0.870; because the external cohort informed model comparison/selection, the external result was secondary/test-influenced and did not enter Scenarios A or B. At the **Figure 3** operating point, whose probability threshold was not reported, sensitivity was 0.800 (exact 95% CI, 0.593–0.932) and specificity 0.773 (0.692–0.841; TP = 20, FN = 5, TN = 102, FP = 30). A separate threshold of 0.29 was optimized in the same external cohort to obtain reported sensitivity 0.88, specificity 0.70, and NPV 0.97; its rounded metrics permit two candidate 2 × 2 tables, so it was retained only as a threshold-sensitivity analysis. No EBV estimates were pooled.

**Figure 3:**
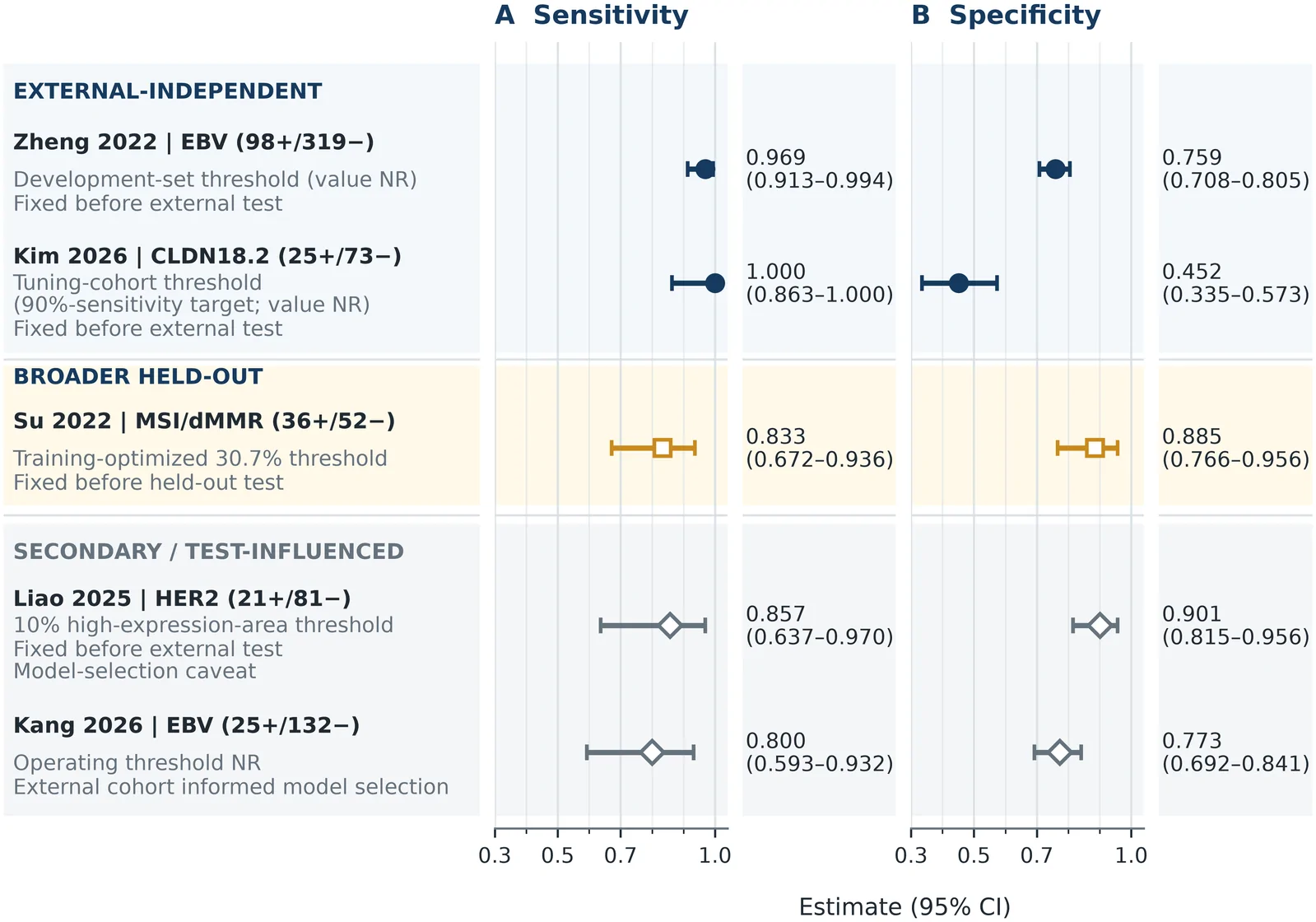
Representative diagnostic-accuracy operating points under the routing framework: **(A)** sensitivity and **(B)** specificity. Estimates are shown with exact binomial 95% CIs; parenthetical +/− counts denote reference-standard-positive/negative cases. The HER2 (42) and EBV (16) operating points are secondary/test-influenced because target-test data informed model or component selection. No pooling was performed. CI, confidence interval; NR, not reported.

### 3.7 HER2 and PD-L1

Two HER2 studies were included (41, 42). Their AUROCs lacked valid sampling uncertainty for the main scenarios. One report provided an external 2 × 2 result (sensitivity 0.857, 95% CI 0.637–0.970; specificity 0.901, 0.815–0.956), but it remained test-set-influenced secondary evidence because external data informed component/model selection. Two studies addressed PD-L1 (7, 40); neither provided an external-independent, patient-level 2 × 2 evaluation, and reference definitions differed. Both markers therefore remained sparse, hypothesis-generating evidence rather than evidence of ineffective prediction.

### 3.8 CLDN18.2

One eligible peer-reviewed H&E-to-CLDN18.2 study (15) was identified. The main external cohort (*n* = 98) yielded AUROC 0.856 (95% CI, 0.775–0.926). At the threshold selected in the tuning cohort to target 90% sensitivity, the external cohort had sensitivity 1.000 (exact 95% CI, 0.863–1.000) and specificity 0.452 (0.335–0.573; TP = 25, FN = 0, TN = 33, FP = 40). The internal cohort yielded AUROC 0.752 (0.702–0.799), sensitivity 0.899 (0.837–0.944), and specificity 0.473 (0.408–0.538) at the same tuning-fixed rule. Cohort-specific Youden thresholds were retained only as threshold-sensitivity analyses. Although encouraging, a single industry-funded study cannot establish generalizability or clinical utility.

## 4 Discussion

This review found that H&E-based models showed potentially useful discrimination for MSI/dMMR and EBV, but reported estimates were frequently dependent because cohorts, models, thresholds, and configurations were reused. Evidence for HER2, PD-L1, and CLDN18.2 remained sparse. High risk of bias and limited external validation further restricted interpretation, leaving the evidence insufficient for clinical deployment.

AUROC alone does not determine sensitivity, specificity, predictive values, the number of confirmatory tests avoided, or the number of biomarker-positive tumors missed at deployment; an H&E-based rule-out or triage role therefore remains a hypothesis. Synthesis was further constrained by heterogeneity in index tests, specimen types, analysis units, reference standards, and validation designs, together with repeated use of TCGA-STAD. Accordingly, no pooled summary, *I*², or τ² was reported. No included study evaluated calibration of the final model, and none reported a reproducible *a priori* sample-size calculation.

The biological premise is nevertheless plausible because H&E does not encode a biomarker molecule directly; it captures tissue phenotypes associated with tumor genotype, cell state, and the microenvironment. Across gastric studies, models have linked MSI/dMMR to differentiation and immune-rich patterns, EBV to the spatial distribution of lymphocytic infiltration, and HER2, PD-L1, or CLDN18.2 to distributed tumor-cell, architectural, stromal, and immune-context features (5–7, 15, 27, 28, 39–42). Computationally, whole-slide images are divided into patches, encoded into multiscale features, and aggregated—often through multiple-instance learning—into slide- or patient-level predictions (5–7, 34). Heatmaps and feature associations can indicate where a model obtains signal, but they establish correlation rather than a causal or marker-specific mechanism. This distinction matters because different biomarkers can share immune-rich morphology, whereas intratumoral heterogeneity can dilute or localize the relevant signal.

Evidence from other organs demonstrates proof of concept, not interchangeability. In colorectal cancer, Echle et al. externally validated H&E-based MSI/dMMR prediction with an AUROC of 0.96 after color normalization (43). In lung adenocarcinoma, Coudray et al. reported held-out AUROCs of 0.733–0.856 for six mutations, although external testing was limited to EGFR and was less accurate (44). These findings show that molecular alterations can have learnable histologic correlates, but the phenotype–label relationship is conditioned by organ architecture, precursor biology, immune and stromal context, specimen type, and laboratory workflow. Gastric and GEJ adenocarcinomas have their own molecular and morphologic organization (45), and recent pan-tissue foundation models demonstrate both transfer across tissues and persistent organ structure in learned representations (46–48). Colon or lung performance therefore cannot validate a gastric test; gastric/GEJ-specific development, calibration, and external multi-institutional validation remain necessary.

Several included studies used pathology-pretrained encoders, including RetCCL and CTransPath; CHIEF and PROGPath provide broader context but do not themselves establish gastric-biomarker performance (5, 30, 33, 49–52). Differences in pretraining may contribute to heterogeneity but cannot be separated from differences in cohorts, tasks, scanners, aggregation, and validation design. The limitations of the present evidence should not be read as a reason to stop development. Larger and more diverse self-supervised pretraining, whole-slide context modeling, and foundation models such as Prov-GigaPath and Virchow have improved transferable pathology representations, while TITAN extends slide-level learning through vision–language alignment with pathology reports (46–48). These advances may improve data efficiency and the capture of weak, spatially distributed phenotypes, so future gastric biomarker models could outperform the studies summarized here. However, better encoders do not correct spectrum bias, cohort reuse, *post hoc* threshold selection, or incomplete reporting; technological progress must be paired with locked evaluation, calibration, and genuinely external validation.

Marker-specific differences are also informative. The sparse and less conclusive PD-L1 evidence should not be interpreted as a meaningless negative result; it may indicate that a spatially heterogeneous, microenvironment-dependent phenotype is only weakly represented by H&E alone or requires context beyond the sampled tissue. This motivates testing multimodal strategies that combine H&E representations with prespecified, routinely available clinicopathologic, radiologic, or molecular data rather than simply optimizing another image-only model. Vision–language foundation models show that pathology images and reports can be aligned at scale (48), and multimodal integration has shown incremental promise for immunotherapy-outcome prediction in lung cancer (53), but neither finding establishes improved gastric PD-L1 prediction. Such models should therefore be treated as distinct index tests, compared head-to-head with H&E-only models, and required to show incremental clinical utility; inputs that are costly or unavailable at triage may undermine the intended low-cost screening role. RSA, DeepComp, HiCervix, FMDet, and SAC-Net address different tasks or modalities and therefore provide methodological context rather than direct evidence for gastric-biomarker prediction (54–58).

Clinical translation should therefore proceed as a staged triage strategy rather than replace confirmatory testing: the intended role, model, and operating threshold should be locked before patient-level, multi-institutional external validation, followed by prospective assessment of false-negative safeguards and clinical utility.

This review has limitations. The estimate-routing hierarchy was finalized during full-text re-extraction rather than prespecified in the protocol, although routing was based on design and dependence characteristics rather than performance magnitude. Restricting synthesis to peer-reviewed full texts may have introduced dissemination bias, and CLDN18.2 was represented by one eligible study. Some report-level relationships and participant or pretraining overlap could not be fully quantified. Sparse independent studies prevented formal assessment of inconsistency and publication bias and precluded subgroup or meta-regression analyses; inability to assess publication bias should not be interpreted as evidence that it was absent. These constraints warrant cautious interpretation of the descriptive performance ranges.

Future studies should predefine the intended clinical role and operating threshold; report exact patient-level 2 × 2 counts with confidence intervals; assess calibration; justify sample size; and undertake external multi-institutional validation in the intended population and specimen type. H&E-only claims should be evaluated using H&E-only inputs, whereas multimodal claims should prespecify the contribution, availability, timing, and cost of each additional modality and quantify incremental value over H&E alone. Gastric/GEJ-specific validation should include biopsy specimens when pretreatment triage is intended and should address staining, scanner, and institutional domain shift. Pretraining provenance and any overlap between pretraining and evaluation cohorts should also be reported explicitly. These steps are necessary before a subsequent DTA meta-analysis or clinical-utility evaluation is meaningful.

In conclusion, H&E-based models showed potentially useful discrimination for MSI/dMMR and EBV, whereas evidence for HER2, PD-L1, and CLDN18.2 remains sparse. Current data are insufficient to support clinical triage, but biologically informed, organ-specific, externally validated development—including multimodal strategies for weak morphology-only signals—remains warranted.

## Data Availability

The original contributions presented in the study are included in the article/**Supplementary Material**. Further inquiries can be directed to the corresponding author.
