# Correction: Deep learning prediction of MSI/dMMR, EBV, HER2, PD-L1, and CLDN18.2 status directly from hematoxylin and eosin-stained slides in gastric and gastroesophageal junction adenocarcinoma: a systematic review

**DOI:** 10.3389/fonc.2026.1999663

**Published:** 2026-09-22

**Authors:** Hyuk Lee, Sang Yun Ha, Sehun Kim, Yang Won Min

**Affiliations:** 1 Department of Medicine, Samsung Medical Center, Sungkyunkwan University School of Medicine, Seoul, Republic of Korea; 2 Department of Pathology and Translational Genomics, Samsung Medical Center, Sungkyunkwan University School of Medicine, Seoul, Republic of Korea; 3 Samsung Precision Genome Medicine Institute, Samsung Medical Center, Seoul, Republic of Korea

**Keywords:** clinical validation, computational pathology, deep learning, diagnostic accuracy, gastric adenocarcinoma, gastroesophageal junction adenocarcinoma, hematoxylin and eosin, molecular biomarkers

In the published article, the parenthetical list markers (11), (12), (1), and (2) in the opening sentence of Section 2.2 were incorrectly treated as references. These four markers, including their citation hyperlinks and bibliography cross-reference tags, should be removed. The genuine in-text citations and reference-list numbering (1–58) should remain unchanged.

A correction has been made to the **2 Methods**, 2.2 *Eligibility criteria*, paragraph 1, opening sentence:

“We included studies that enrolled patients with gastric or GEJ adenocarcinoma; used artificial intelligence (AI)/DL applied to H&E-stained whole-slide or tissue-microarray images as the index test; predicted MSI/dMMR, EBV, HER2, PD-L1, or CLDN18.2 status; and used a molecular, IHC, or *in situ* hybridization reference standard.”

In the published article, affiliation 3, “Samsung Precision Genome Medicine Institute, Samsung Medical Center, Seoul, Republic of Korea,” was omitted for Hyuk Lee, Sang Yun Ha, and Yang Won Min. The correct affiliations are Hyuk Lee: 1 and 3; Sang Yun Ha: 2 and 3; and Yang Won Min: 1 and 3. Sehun Kim remains affiliated with affiliation 3 only. The institution names, affiliation numbering, equal-contribution designations, and corresponding-author designation remain unchanged.

The original version of this article has been updated.

