# Supplementary material for: Deep learning prediction of MSI/dMMR, EBV, HER2, PD-L1, and CLDN18.2 status directly from hematoxylin and eosin-stained slides in gastric and gastroesophageal junction adenocarcinoma: a systematic review
Source: Front Oncol. 2026 Sep 9;16:1953562. doi: 10.3389/fonc.2026.1953562 (PMC13651465; doi:10.3389/fonc.2026.1953562)
Supplement: Supplementary file 1 [file Supplementaryfile1.docx]

Supplementary Material

Supplementary Methods and Search Audit

# 1 Information sources and search accounting

The original searches for the four prespecified biomarkers (MSI/dMMR, EBV, HER2, and PD-L1) were executed on database-specific dates between July 14 and July 26, 2026, with coverage from database inception (PubMed, July 14-26; Embase and CENTRAL, July 15; Web of Science Core Collection, July 26). A four-marker PubMed update covering July 27 through August 6, 2026 retrieved 44 raw records; one within-update duplicate was removed, and all 43 unique records were excluded at title/abstract. On August 6, 2026, CLDN18.2 was added as a reviewer-prompted protocol amendment; the amendment was incorporated into the PROSPERO record (CRD420261449992) on August 7, 2026, without relabeling CLDN18.2 as an originally prespecified target. The focused search was executed in PubMed on August 6 (1 hit) and in Embase (5 hits), CENTRAL (5 reproducible hits), and Web of Science Core Collection (2 hits) on August 7. Across these four databases, 13 raw records yielded 9 unique reports after removal of 4 duplicates; 8 were excluded at title/abstract, and Kim et al. (47) was retained for formal synthesis. An exact retrospective rerun of the same PubMed update queries on August 8 returned 56 raw occurrences and 53 unique records. Relative to the locked August 6 output, the late-indexing increment comprised 12 raw occurrences, 2 within-batch duplicate occurrences, and 10 unique records; none was a global duplicate of prior database material. Nine were excluded at title/abstract, and Kang et al. (60) was included after full-text assessment. YWM and HL independently confirmed the screening, extraction, routing, risk-of-bias, and certainty judgments for the added evidence; no disagreement required SYH adjudication. The reconciled search arithmetic is final.

| **Source / interface** | **Search date** | **Search component** | **Raw hits** |
| --- | --- | --- | --- |
| PubMed (NCBI) | 2026-07-14 to 2026-07-26 | Original accepted PubMed searches | 762 |
| Embase | 2026-07-15 | Original accepted Emtree/free-text query | 125 |
| Cochrane CENTRAL | 2026-07-15 | Original accepted free-text query | 93 |
| Web of Science Core Collection | 2026-07-26 | Original accepted topic search | 45 |
| PubMed (NCBI) | 2026-07-27 to 2026-08-06 | Four-marker high-recall update; no eligible study | 44 |
| PubMed (NCBI) | 2026-08-06 | Focused CLDN18.2 search; included Kim et al. (47) | 1 |
| Embase | 2026-08-07 | Focused CLDN18.2 amendment search; no additional eligible study | 5 |
| Cochrane CENTRAL | 2026-08-07 | Exact focused CLDN18.2 rerun; all excluded at title/abstract | 5 |
| Web of Science Core Collection | 2026-08-07 | Focused CLDN18.2 amendment search; no additional eligible study | 2 |
| PubMed (NCBI) | 2026-08-08 | Late-indexing rerun: 12 raw, 2 within-batch duplicates, 10 unique; included Kang et al. (60) | 12 |
| Final total | through 2026-08-08 | All executed identification searches | 1,094 |

For the original five PubMed update queries, individual counts summed to 76 and their within-round deduplicated union was 55. The July 27-August 6, 2026 update yielded 44 raw and 43 unique records, all excluded at title/abstract. The exact August 8 rerun identified a separate late-indexing increment without altering the locked 43-record workbook: 12 raw occurrences, 2 within-batch duplicates, and 10 unique records; nine were excluded and the late-indexed EBV study was included. The focused CLDN18.2 searches yielded 13 raw and 9 unique reports after 4 duplicates; the CLDN18.2 study was the sole eligible report. Documentation-only coverage checks, exact-title/DOI searches, and companion tracing were not added as independent records.

| **Selection stage** | **Count** |
| --- | --- |
| Records identified from databases | 1,094 |
| Duplicate records removed | 393 |
| Unique records screened | 701 |
| Records excluded at title/abstract | 664 |
| Full-text reports assessed | 37 |
| Full-text reports excluded | 9 |
| Peer-reviewed full-text primary reports included | 28 |
| Focused CLDN18.2 records (raw / duplicates / unique screened / excluded / included) | 13 / 4 / 9 / 8 / 1; included: Kim et al. (47) |
| Late-indexing increment (raw / within-batch duplicates / unique screened / title-abstract excluded / included) | 12 / 2 / 10 / 9 / 1; included: Kang et al. (60) |

The final reconciled flow is 1,094 records identified, 393 duplicates removed, 701 records screened, 664 title/abstract exclusions, 37 full texts assessed, nine full-text exclusions, and 28 included peer-reviewed full-text primary reports. The original full-text exclusion reasons remain unchanged. The arithmetic checks are 1,094 - 393 = 701, 701 - 664 = 37, and 37 - 9 = 28.

# 2 Full electronic search strategies

The strings below reproduce the executed searches available in the retained audit materials. The search-accounting table records the four-marker PubMed update, the reviewer-prompted CLDN18.2 searches in PubMed, Embase, CENTRAL, and Web of Science Core Collection, and the August 8 exact PubMed rerun used to isolate the late-indexing increment.

## 2.1 PubMed initial searches (July 14, 2026)

MSI/dMMR (hits: 87)

(gastric OR stomach OR gastroesophageal OR esophagogastric) AND ("deep learning" OR "machine learning" OR "artificial intelligence" OR "convolutional neural network" OR "computational pathology" OR "digital pathology") AND ("microsatellite instability" OR MSI OR "mismatch repair" OR dMMR)

EBV (hits: 33)

(gastric OR stomach OR gastroesophageal OR esophagogastric) AND ("deep learning" OR "machine learning" OR "artificial intelligence" OR "convolutional neural network" OR "computational pathology" OR "digital pathology") AND ("Epstein-Barr" OR EBV OR "Epstein Barr virus")

HER2 (hits: 53)

(gastric OR stomach OR gastroesophageal OR esophagogastric) AND ("deep learning" OR "machine learning" OR "artificial intelligence" OR "convolutional neural network" OR "computational pathology" OR "digital pathology") AND (HER2 OR ERBB2 OR "human epidermal growth factor receptor 2")

PD-L1 (hits: 61)

(gastric OR stomach OR gastroesophageal OR esophagogastric) AND ("deep learning" OR "machine learning" OR "artificial intelligence" OR "convolutional neural network" OR "computational pathology" OR "digital pathology") AND ("PD-L1" OR PDL1 OR "programmed death ligand" OR "programmed cell death ligand")

## 2.2 Embase (July 15, 2026)

Accepted Emtree and free-text search (hits: 125)

('stomach tumor'/exp OR 'stomach adenocarcinoma'/exp OR gastric:ti,ab OR 'gastroesophageal junction':ti,ab OR 'gastro-oesophageal junction':ti,ab OR 'esophagogastric junction':ti,ab OR GEJ:ti,ab) AND ('deep learning'/exp OR 'machine learning'/exp OR 'artificial intelligence'/exp OR 'convolutional neural network'/exp OR 'digital pathology'/exp OR 'computational pathology':ti,ab OR 'whole slide':ti,ab OR 'whole-slide':ti,ab OR histopatholog*:ti,ab OR hematoxylin:ti,ab OR haematoxylin:ti,ab OR 'h&e':ti,ab OR pathomic*:ti,ab) AND ('microsatellite instability'/exp OR MSI:ti,ab OR 'mismatch repair deficiency'/exp OR dMMR:ti,ab OR 'Epstein Barr virus'/exp OR EBV:ti,ab OR EBER:ti,ab OR 'epidermal growth factor receptor 2'/exp OR HER2:ti,ab OR ERBB2:ti,ab OR 'programmed death 1 ligand 1'/exp OR 'PD-L1':ti,ab OR CD274:ti,ab)

## 2.3 Cochrane CENTRAL (July 15, 2026)

CENTRAL free-text search (hits: 93)

(gastric OR stomach OR gastroesophageal OR gastro-oesophageal OR esophagogastric OR GEJ) AND ("deep learning" OR "machine learning" OR "artificial intelligence" OR "convolutional neural" OR "whole slide" OR histopatholog* OR hematoxylin OR "H&E") AND ("microsatellite instability" OR MSI OR "mismatch repair" OR "Epstein-Barr" OR EBV OR HER2 OR ERBB2 OR "PD-L1" OR CD274)

## 2.4 Web of Science Core Collection (July 26, 2026)

SCI-EXPANDED and CPCI-S topic search (hits: 45)

TS=((gastric OR stomach OR gastroesophageal OR "gastro-oesophageal" OR esophagogastric) AND ("deep learning" OR "machine learning" OR "artificial intelligence" OR convolutional OR "neural network" OR transformer OR "multiple instance" OR "weakly supervised") AND (histopatholog* OR "whole slide" OR "whole-slide" OR WSI OR "H&E" OR hematoxylin OR haematoxylin OR pathomic*) AND (microsatellite OR MSI OR "mismatch repair" OR dMMR OR "Epstein-Barr" OR EBV OR EBER OR HER2 OR ERBB2 OR "PD-L1" OR CD274))

## 2.5 PubMed supplementary and high-recall searches (July 26, 2026)

Method-vocabulary supplement (hits: 4)

gastric[Title/Abstract] AND (foundation model[Title/Abstract] OR self-supervised[Title/Abstract] OR transformer[Title/Abstract] OR multiple instance[Title/Abstract] OR weakly supervised[Title/Abstract]) AND (microsatellite[Title/Abstract] OR "Epstein-Barr"[Title/Abstract] OR HER2[Title/Abstract] OR "PD-L1"[Title/Abstract])

MeSH recall search (hits: 72)

"Stomach Neoplasms"[MeSH] AND "Deep Learning"[MeSH] AND (pathology[Title/Abstract] OR histolog*[Title/Abstract] OR "whole slide"[Title/Abstract] OR hematoxylin[Title/Abstract])

High-recall sweep A (MSI/EBV) (hits: 161)

(gastric[Title/Abstract] OR esophagogastric[Title/Abstract]) AND ("whole slide"[Title/Abstract] OR histopatholog*[Title/Abstract] OR hematoxylin[Title/Abstract] OR pathology image*[Title/Abstract]) AND (microsatellite[Title/Abstract] OR "Epstein-Barr"[Title/Abstract] OR EBV[Title/Abstract])

High-recall sweep B (HER2/PD-L1) (hits: 215)

(gastric[Title/Abstract] OR esophagogastric[Title/Abstract]) AND ("whole slide"[Title/Abstract] OR histopatholog*[Title/Abstract] OR hematoxylin[Title/Abstract] OR pathology image*[Title/Abstract]) AND (HER2[Title/Abstract] OR ERBB2[Title/Abstract] OR "PD-L1"[Title/Abstract] OR CD274[Title/Abstract])

The two high-recall sweeps intentionally omitted a separate methodological concept block. They were used as supplementary recall searches, not as the sole reproducible search strategy.

## 2.6 PubMed update searches (July 26, 2026)

Because the search interface imposed a Boolean-operator limit, the update was executed as five marker/wording-specific queries. Their deduplicated union contained 55 records.

Common block POP (hits: definition)

("Stomach Neoplasms"[Mesh] OR gastric[tiab] OR gastroesophageal[tiab] OR esophagogastric[tiab])

Common block MET (hits: definition)

("Artificial Intelligence"[Mesh] OR "deep learning"[tiab] OR "machine learning"[tiab] OR convolutional[tiab] OR "neural network"[tiab] OR "multiple instance"[tiab])

Common block IMG (hits: definition)

(histopathology[tiab] OR histopathological[tiab] OR histology[tiab] OR histological[tiab] OR "whole slide"[tiab] OR hematoxylin[tiab])

Common block IMG+TMA (hits: definition)

(histopathology[tiab] OR histopathological[tiab] OR histology[tiab] OR histological[tiab] OR "whole slide"[tiab] OR hematoxylin[tiab] OR "tissue microarray"[tiab])

Update 1: MSI/dMMR (hits: 20)

("Stomach Neoplasms"[Mesh] OR gastric[tiab] OR gastroesophageal[tiab] OR esophagogastric[tiab]) AND ("Artificial Intelligence"[Mesh] OR "deep learning"[tiab] OR "machine learning"[tiab] OR convolutional[tiab] OR "neural network"[tiab] OR "multiple instance"[tiab]) AND (histopathology[tiab] OR histopathological[tiab] OR histology[tiab] OR histological[tiab] OR "whole slide"[tiab] OR hematoxylin[tiab]) AND ("Microsatellite Instability"[Mesh] OR microsatellite[tiab] OR MSI[tiab] OR "mismatch repair"[tiab] OR dMMR[tiab])

Update 2: EBV (hits: 11)

("Stomach Neoplasms"[Mesh] OR gastric[tiab] OR gastroesophageal[tiab] OR esophagogastric[tiab]) AND ("Artificial Intelligence"[Mesh] OR "deep learning"[tiab] OR "machine learning"[tiab] OR convolutional[tiab] OR "neural network"[tiab] OR "multiple instance"[tiab]) AND (histopathology[tiab] OR histopathological[tiab] OR histology[tiab] OR histological[tiab] OR "whole slide"[tiab] OR hematoxylin[tiab]) AND ("Herpesvirus 4, Human"[Mesh] OR "Epstein-Barr"[tiab] OR EBV[tiab] OR EBER[tiab])

Update 3: HER2 (hits: 5)

("Stomach Neoplasms"[Mesh] OR gastric[tiab] OR gastroesophageal[tiab] OR esophagogastric[tiab]) AND ("Artificial Intelligence"[Mesh] OR "deep learning"[tiab] OR "machine learning"[tiab] OR convolutional[tiab] OR "neural network"[tiab] OR "multiple instance"[tiab]) AND (histopathology[tiab] OR histopathological[tiab] OR histology[tiab] OR histological[tiab] OR "whole slide"[tiab] OR hematoxylin[tiab] OR "tissue microarray"[tiab]) AND ("Receptor, ErbB-2"[Mesh] OR HER2[tiab] OR ERBB2[tiab])

Update 4: PD-L1 (hits: 5)

("Stomach Neoplasms"[Mesh] OR gastric[tiab] OR gastroesophageal[tiab] OR esophagogastric[tiab]) AND ("Artificial Intelligence"[Mesh] OR "deep learning"[tiab] OR "machine learning"[tiab] OR convolutional[tiab] OR "neural network"[tiab] OR "multiple instance"[tiab]) AND (histopathology[tiab] OR histopathological[tiab] OR histology[tiab] OR histological[tiab] OR "whole slide"[tiab] OR hematoxylin[tiab] OR "tissue microarray"[tiab]) AND ("B7-H1 Antigen"[Mesh] OR "PD-L1"[tiab] OR CD274[tiab])

Update 5: broad pathology wording (hits: 35)

("Stomach Neoplasms"[Mesh] OR gastric[tiab] OR gastroesophageal[tiab] OR esophagogastric[tiab]) AND ("Artificial Intelligence"[Mesh] OR "deep learning"[tiab] OR "machine learning"[tiab] OR convolutional[tiab] OR "neural network"[tiab] OR "multiple instance"[tiab]) AND (pathology[tiab] OR pathological[tiab] OR pathologist[tiab]) AND (microsatellite[tiab] OR MSI[tiab] OR "Epstein-Barr"[tiab] OR EBV[tiab] OR HER2[tiab] OR "PD-L1"[tiab])

## 2.7 Reviewer-prompted CLDN18.2 protocol amendment (August 6-7, 2026)

Executed focused PubMed query (August 6, 2026; hits: 1; eligible peer-reviewed full text: Kim et al. (47))

(gastric[Title/Abstract] OR stomach[Title/Abstract] OR gastroesophageal[Title/Abstract] OR esophagogastric[Title/Abstract]) AND (histolog*[Title/Abstract] OR histopatholog*[Title/Abstract] OR "whole slide"[Title/Abstract] OR "whole-slide"[Title/Abstract] OR H&E[Title/Abstract] OR hematoxylin[Title/Abstract] OR haematoxylin[Title/Abstract]) AND ("deep learning"[Title/Abstract] OR "machine learning"[Title/Abstract] OR "artificial intelligence"[Title/Abstract] OR transformer[Title/Abstract] OR "multiple instance"[Title/Abstract]) AND (CLDN18.2[Title/Abstract] OR "claudin 18.2"[Title/Abstract] OR CLDN18[Title/Abstract] OR "claudin 18"[Title/Abstract])

Across the four focused database searches, 13 raw records yielded 9 unique reports after removal of 4 duplicates. Eight were excluded at title/abstract, and Kim et al. (47) was the sole report retained for formal synthesis. Documentation-only high-recall CLDN coverage (103 hits) and a method-focused cross-check (18 hits) were screened for coverage and companion tracing but were not added as independent identification records.

The ASCO 2025 abstract (J Clin Oncol 43, abstract 4047; doi: 10.1200/JCO.2025.43.16_suppl.4047) was linked to Kim et al. 2026 as a companion report of the same evolving study. It was retained for tracing only and was not counted as a separate study or estimate.

Evidence-governance note: YWM and HL independently confirmed the focused-search screening and primary exclusion reasons, extraction and routing of the CLDN18.2 study, its three QUADAS-2/PROBAST scope assessments, and its GRADE judgment. They also confirmed the late-indexed batch screening, nine title/abstract exclusion reasons, eligibility, extraction, and routing of the EBV study, its two risk-of-bias scopes, and the EBV GRADE update. No disagreement required SYH adjudication. These confirmations support the final reconciled search arithmetic.

**Executed focused CLDN18.2 database searches (August 6-7, 2026)**

Embase (executed August 7, 2026; hits: 5): ('stomach tumor'/exp OR 'stomach adenocarcinoma'/exp OR gastric:ti,ab OR 'gastroesophageal junction':ti,ab OR esophagogastric:ti,ab) AND ('histopathology'/exp OR 'whole slide':ti,ab OR histolog*:ti,ab OR hematoxylin:ti,ab OR haematoxylin:ti,ab OR 'h&e':ti,ab) AND ('artificial intelligence'/exp OR 'deep learning'/exp OR 'machine learning'/exp OR transformer:ti,ab OR 'multiple instance':ti,ab) AND ('claudin 18.2':ti,ab OR cldn18.2:ti,ab OR cldn18:ti,ab OR 'claudin 18':ti,ab)

CENTRAL (exact rerun executed August 7, 2026; hits: 5): (gastric OR stomach OR gastroesophageal OR esophagogastric) AND (histolog* OR histopatholog* OR "whole slide" OR hematoxylin OR haematoxylin OR "H&E") AND ("deep learning" OR "machine learning" OR "artificial intelligence" OR transformer OR "multiple instance") AND (CLDN18.2 OR "claudin 18.2" OR CLDN18 OR "claudin 18")

Web of Science Core Collection (executed August 7, 2026; SCI-EXPANDED and CPCI-S; hits: 2): TS=((gastric OR stomach OR gastroesophageal OR esophagogastric) AND (histolog* OR histopatholog* OR "whole slide" OR hematoxylin OR haematoxylin OR "H&E") AND ("deep learning" OR "machine learning" OR "artificial intelligence" OR transformer OR "multiple instance") AND (CLDN18.2 OR "claudin 18.2" OR CLDN18 OR "claudin 18"))

CENTRAL count audit: The exact documented rerun returned five CENTRAL Trials. An earlier manual note of six hits could not be reproduced, and search terms were not altered to force that count. The auditable count used throughout the submission is therefore five; the six-hit note is retained only as a discrepancy record.

## 2.8 Four-marker update (July 27-August 6, 2026)

Marker-specific focused PubMed date-window queries covering July 27-August 6, 2026 yielded zero records for MSI/dMMR, EBV, HER2, and PD-L1. Three high-recall checks yielded the locked August 6 output of 44 raw records (43 unique after one within-update duplicate); all were excluded at title/abstract. The exact August 8 rerun returned 56 raw occurrences and 53 unique records. The late-indexing increment comprised 12 raw occurrences, 10 unique records, and 2 within-batch duplicates; none of the 10 was a global duplicate of existing database material. Nine were excluded at title/abstract, and the late-indexed EBV study was included after full-text assessment. This was treated as indexing lag rather than a missed historical screening decision, and the historical 44/43 workbook remained unchanged.

# 3 Evidence classification and routing (version 2)

Validation_class_v2: external-independent, external-public, same-institution patient-disjoint heldout, same-institution specimen/scanner shift, cross-validation/resampling, development/tuning, or unclear/mixed. Analysis_unit_v2: patient, specimen/slide, tile/ROI, or mixed/unclear. Patient_separation_v2: documented or not documented. Reference_standard_v2: clinically anchored, mixed, research surrogate, or unclear/NR. Display_route_v2: main external, broader heldout, dependence-aware, threshold sensitivity, test-influenced secondary, narrative, or out-of-scope. Target-test influence was captured separately in Test_influence_v2 and did not define the validation setting.

Outcome-blind decision sequence: eligibility -> analysis unit and patient separation -> validation setting -> reference-standard class -> metric verifiability -> threshold timing -> cohort/model overlap -> tie-breakers favoring greater externality, patient-level evaluation, a clinically anchored reference, no test-set influence, the report's primary configuration, and then larger sample size. Scenario A requires genuinely external, patient-level, clinically anchored evaluation without adaptation. Scenario B adds same-institution patient-disjoint held-out evaluation.

Kang et al. (60), a peer-reviewed EBV biopsy study published online in J Pathol on August 5, 2026 (doi: 10.1002/path.70105), reported an external DSMIL-MB AUROC of 0.870. This result was retained as test-influenced secondary evidence and excluded from the main and broader held-out counts. The exact external operating point was TP = 20, FN = 5, TN = 102, and FP = 30; the threshold was not reported. A separate threshold of 0.29, selected and evaluated in the external EWUM cohort, was retained only for threshold-sensitivity analysis because its rounded metrics did not uniquely determine exact counts.

The study contributed two risk-of-bias scopes: internal patient-level five-fold cross-validation and external EWUM validation. Both were QUADAS-2 High overall (D1 High, D2 High, D3 Low, D4 Unclear; applicability concerns Low) and PROBAST High overall (Participants High, Predictors Low, Outcome Low, Analysis High). The EBV evidence body therefore comprises 14 studies and remains Very low certainty; the main external AUROC, broader held-out AUROC, and main/broader DTA counts were unchanged.

Conference abstracts and preprints are excluded from the formal numerical synthesis and retained only for subsequent-publication and companion-report tracing. When a linked eligible peer-reviewed full-text primary report exists, only that full-text report contributes to formal synthesis. This choice improves verifiability but may introduce dissemination bias and does not imply that peer review removes risk of bias.

# 4 Search-audit notes and protocol deviations

• Scopus and IEEE Xplore were not searched because institutional access was unavailable. Web of Science Core Collection, including SCI-EXPANDED and CPCI-S, was searched to broaden journal and conference-proceedings coverage.

• PROSPERO section D14 specified that medRxiv, arXiv, and bioRxiv records would be screened separately and flagged. No executed preprint-server search was documented in the retained search log; this is a protocol deviation. Conference abstracts and preprints were excluded from formal synthesis and retained only for subsequent-publication and companion-report tracing; when a linked eligible peer-reviewed full-text report existed, only the full-text report contributed.

• The July 26 supplementary PubMed queries were retrospectively reconstructed from retained session and workbook records rather than a contemporaneously completed log. The July 27-August 6, 2026 PubMed update and the August 6-7 CLDN18.2 searches were documented at execution.

• A broad Embase formulation (1,530 hits) and two PubMed alternatives (23 and 174 hits) were rejected during original strategy development because of poor recall or excessive off-target retrieval. They contributed no records to the PRISMA identification count.

• The CLDN18.2 addition was a dated reviewer-prompted amendment, not an originally prespecified target. The amendment was subsequently incorporated into the PROSPERO record (CRD420261449992) on August 7, 2026. The focused PubMed, Embase, CENTRAL, and Web of Science searches were completed and globally deduplicated. The exact CENTRAL rerun returned five records; an earlier six-hit note was not reproducible and is retained only as an audit discrepancy, not in the submission count.

• The exact August 8 PubMed rerun isolated 12 late-indexed raw occurrences, 2 within-batch duplicates, and 10 globally unique records. Nine were excluded at title/abstract, and the report by Kang et al. (60) was included after full-text assessment. This separate audit did not overwrite the locked August 6 historical screening workbook.

# 5 Supplementary data tables

The accompanying Supplementary Data workbook contains the following auditable tables:

• Included_Studies: 28 report-level records, including Kim et al. (47) for CLDN18.2 and Kang et al. (60) for EBV prediction from gastric biopsy images, with cohort sizes, reference standards, analysis units, and funding statements.

• AUROC_Results: 204 rows (197 original, five CLDN18.2, and two from the EBV study: internal DSMIL-MB 0.907 ± 0.044 and external DSMIL-MB 0.870), with validation class, analysis unit, reference-standard class, threshold timing, dependence, Scope ID, and display route. Its external AUROC was classified as test-influenced secondary evidence.

• DTA_2x2: 40 exact or uniquely recoverable threshold-specific rows (35 original, four CLDN18.2, and the EBV study's exact external operating point), with exact confidence intervals and threshold timing. For that study, TP = 20, FN = 5, TN = 102, and FP = 30; the threshold was not reported. The 0.29 threshold was retained only as a sensitivity-analysis configuration because same-cohort optimization and rounded metrics did not yield unique exact counts.

• RoB_Scopes: 42 analytic scopes (37 original, three CLDN18.2, and two from the EBV study: internal patient-level five-fold cross-validation and external EWUM validation), with QUADAS-2 and PROBAST domain rationales and overall judgments. QUADAS-2 risk was High for 40 scopes and Unclear for 2; PROBAST risk was High for all 42. Both scopes were QUADAS-2 High overall (D1 High, D2 High, D3 Low, D4 Unclear; applicability concerns Low) and PROBAST High overall (Participants High, Predictors Low, Outcome Low, Analysis High).

• GRADE_SoF: separate certainty judgments for MSI/dMMR, EBV, HER2, PD-L1, and CLDN18.2. EBV now includes 14 studies and remains Very low certainty; this addition did not change the main external AUROC count (2), broader held-out AUROC count (3), or main/broader DTA report count (1). Certainty was Very low for MSI/dMMR, EBV, HER2, and PD-L1 and Low for CLDN18.2; the CLDN18.2 judgment reflected serious risk of bias and imprecision without an indirectness downgrade.

• Search_Accounting, Routing_Dictionary, and Study_Cohort_Technical: final source counts, including the separately audited late-indexing increment; the version-2 routing algorithm and definitions; and report-marker-cohort-model technical fields, including encoder pretraining and possible evaluation overlap.

Abbreviations: AUROC, area under the receiver operating characteristic curve; CENTRAL, Cochrane Central Register of Controlled Trials; CLDN18.2, claudin 18 isoform 2; dMMR, mismatch-repair deficiency; DTA, diagnostic test accuracy; EBV, Epstein-Barr virus; GEJ, gastroesophageal junction; H&E, hematoxylin and eosin; HER2, human epidermal growth factor receptor 2; MSI, microsatellite instability; PD-L1, programmed death-ligand 1; PRISMA, Preferred Reporting Items for Systematic Reviews and Meta-Analyses; PROBAST, Prediction model Risk Of Bias ASsessment Tool; QUADAS-2, Quality Assessment of Diagnostic Accuracy Studies 2; TMA, tissue microarray; WSI, whole-slide image.
