# Supplementary material for: Deep learning prediction of MSI/dMMR, EBV, HER2, PD-L1, and CLDN18.2 status directly from hematoxylin and eosin-stained slides in gastric and gastroesophageal junction adenocarcinoma: a systematic review
Source: Front Oncol. 2026 Sep 9;16:1953562. doi: 10.3389/fonc.2026.1953562 (PMC13651465; doi:10.3389/fonc.2026.1953562)
Supplement: Supplementary file 2 [file Supplementaryfile3.docx]

**PRISMA-DTA 2018 Checklist**

**This v14 checklist is submission-ready. CONTENT LOCATIONS VERIFIED. This checklist incorporates the final late-indexing reconciliation (1,094 identified; 393 duplicates; 701 screened; 664 title/abstract exclusions; 37 full texts; 9 full-text exclusions; 28 included). Ten unique late-indexing records comprised 9 title/abstract exclusions and the formally included EBV report by Kang et al. (60), adding 2 risk-of-bias scopes: 42 scopes across 28 reports overall (QUADAS-2: 40 High, 2 Unclear; PROBAST: 42 High). Conference abstracts and preprints remain limited to subsequent-publication and companion-report tracing and do not contribute to formal synthesis. Locations are reported by section, table, figure, and supplementary item.**

| **Section / Topic** | **No** | **Checklist item** | **Reported location** |
| --- | --- | --- | --- |
| **Title** | | | |
| Title | 1 | Identify the report as a systematic review (+/- meta-analysis) of diagnostic test accuracy (DTA) studies. | Title page |
| **Abstract** | | | |
| Structured summary | 2 | Provide a structured summary including, as applicable, background, objectives, data sources, study eligibility criteria, participants, index tests, reference standard, appraisal and synthesis methods; results (number of studies and summary estimates of diagnostic accuracy); limitations; conclusions and implications of key findings; funding; systematic review registration number with the name of the registry. | Abstract |
| **Background** | | | |
| Rationale | 3 | Describe the rationale for the review in the context of what is already known. | Introduction |
| Clinical role of index test | D1 | State the scientific and clinical background, including the intended use and clinical role of the index test, and if applicable, the rationale for minimally acceptable test accuracy (or minimum difference in accuracy for comparative design). | Introduction; Discussion |
| Objectives | 4 | Provide an explicit statement of question(s) being addressed in terms of participants, index tests, and target conditions. | Introduction |
| **Methods** | | | |
| Protocol and registration | 5 | Indicate if a review protocol exists, if and where it can be accessed, and, if available, provide registration information including registration number. | Methods - Protocol and registration |
| Eligibility criteria | 6 | Specify study characteristics (participants, setting, index tests, reference standards, target conditions, and study design) and report characteristics (e.g., years considered, language, publication status) used as criteria for eligibility, giving rationale. | Methods - Eligibility criteria |
| Information sources | 7 | Describe all information sources (e.g., databases with dates of coverage, contact with study authors to identify additional studies) in the search and date last searched. | Methods - Information sources and search; Supplementary Material 1, including late-indexing reconciliation |
| Search | 8 | Present full search strategies for all electronic databases and other sources searched, including any limits used, such that they could be repeated. | Methods - Information sources and search; Supplementary Material 1, all executed strategies and counts |
| Study selection | 9 | State the process for selecting studies (i.e., screening, eligibility, included in systematic review, and, if applicable, included in the meta-analysis). | Methods - Study selection and data extraction, including late-indexing screening |
| Data collection process | 10 | Describe method of data extraction from reports (e.g., piloted forms, independently, in duplicate) and any processes for obtaining and confirming data from investigators. | Methods - Study selection and data extraction |
| Definitions for data extraction | 11 | Provide definitions used in data extraction and classifications of target condition(s), index test(s), reference standard(s) and other characteristics (e.g. study design, clinical setting). | Methods; Table 1; Supplementary Material 2, Included_Studies, Routing_Dictionary, and Study_Cohort_Technical |
| Risk of bias and applicability | 12 | Describe methods used for assessing risk of bias in individual studies and concerns regarding the applicability to the review question. | Methods - Risk-of-bias assessment; Supplementary Material 2, RoB_Scopes |
| Diagnostic accuracy measures | 13 | State the principal diagnostic accuracy measure(s) reported (e.g. sensitivity, specificity) and state the unit of assessment (e.g. per-patient, per-lesion). | Methods - Data synthesis; Supplementary Material 2, AUROC_Results and DTA_2x2 |
| Synthesis of results | 14 | Describe methods of handling data, combining results of studies and describing variability between studies. This could include, but is not limited to: a) handling of multiple definitions of target condition, b) handling of multiple thresholds of test positivity, c) handling multiple index test readers, d) handling of indeterminate test results, e) grouping and comparing tests, f) handling of different reference standards. | Methods - Data synthesis; Supplementary Material 1 - Routing framework |
| Meta-analysis | D2 | Report the statistical methods used for meta-analyses, if performed. | Not applicable - no statistical meta-analysis; Methods - Data synthesis |
| Additional analyses | 16 | Describe methods of additional analyses (e.g., sensitivity or subgroup analyses, meta-regression), if done, indicating which were pre-specified. | Methods - Protocol deviations and Data synthesis; Supplementary Material 1 |
| **Results** | | | |
| Study selection | 17 | Provide numbers of studies screened, assessed for eligibility, included in the review (and included in meta-analysis, if applicable) with reasons for exclusions at each stage, ideally with a flow diagram. | Results - Study selection; Figure 1 legend: 1,094 identified / 393 duplicates / 701 screened / 664 title-abstract exclusions / 37 full texts / 9 full-text exclusions / 28 included; 10 unique late-indexing records = 9 title-abstract exclusions + Kang et al. (60) included |
| Study characteristics | 18 | For each included study provide citation and present key characteristics including: a) participant descriptions, b) clinical setting, c) study design, d) target condition definition(s), e) index test(s), f) reference standard(s), g) sample size, h) funding sources. | Results - Study characteristics; Table 1; Supplementary Material 2, Included_Studies and Study_Cohort_Technical (28 included reports; Kang et al. (60) is the added EBV report) |
| Risk of bias and applicability | 19 | Present evaluation of risk of bias and concerns regarding applicability for each study. | Results - Risk of bias and applicability; Table 2; Supplementary Material 2, RoB_Scopes (42 scopes/28 reports; QUADAS-2: 40 High, 2 Unclear; PROBAST: 42 High) |
| Results of individual studies | 20 | For each analysis, report the numbers of participants and, if possible, 2×2 tables, forest plots and/or estimates of accuracy and their precision (e.g. sensitivity and specificity), ideally with a forest or SROC plot. | Results - Marker-specific results; Figures 2-3 legends; Supplementary Material 2, AUROC_Results and DTA_2x2 |
| Synthesis of results | 21 | Describe test accuracy, including variability; if meta-analysis was done, include results and confidence intervals. | Results - Marker-specific results; Figures 2-3 legends; no pooled estimate was calculated |
| Additional analyses | 23 | Give results of additional analyses, if done (e.g., sensitivity or subgroup analyses, meta-regression; analysis of index test: failure rates, proportion of inconclusive results, adverse events). | Methods - Data synthesis; Results - Marker-specific results; alternative routing and reference-standard fields in Supplementary Material 2 |
| **Discussion** | | | |
| Summary of evidence | 24 | Summarise the main findings including the strength of evidence. | Results - Certainty of evidence; Discussion; Supplementary Material 2, GRADE_SoF |
| Limitations | 25 | Discuss limitations from included studies (e.g. risk of bias and concerns regarding applicability) and from the review process (e.g. incomplete retrieval of identified research, reporting bias). | Discussion |
| Conclusions | 26 | Provide a general interpretation of the results in the context of other evidence. Discuss implications for future research and clinical practice (e.g. the intended use and clinical role of the index test). | Discussion, including conclusion |
| **Funding** | | | |
| Funding | 27 | For the systematic review, describe the sources of funding and other support and the role of the funders. | Funding |
